# Array-based genome-wide RNAi screening to identify shRNAs that enhance p53-related apoptosis in human cancer cells

**DOI:** 10.18632/oncotarget.2272

**Published:** 2014-07-27

**Authors:** Masashi Idogawa, Tomoko Ohashi, Jun Sugisaka, Yasushi Sasaki, Hiromu Suzuki, Takashi Tokino

**Affiliations:** ^1^ Department of Medical Genome Sciences, Research Institute for Frontier Medicine, Sapporo Medical University School of Medicine, Sapporo, Japan; ^2^ Department of Molecular Biology, Sapporo Medical University School of Medicine, Sapporo, Japan

**Keywords:** p53, shRNA library, apoptosis, PRIMA-1

## Abstract

p53 transduction is a potentially effective cancer therapy but does not result in a good therapeutic response in all human cancers due to resistance to apoptosis. To discover factors that overcome resistance to p53-induced apoptosis, we attempted to identify RNAi sequences that enhance p53-induced apoptosis. We screened a genome-wide lentiviral shRNA library in liver cancer Huh-7 and pancreatic cancer Panc-1 cells, both of which resist p53-induced apoptosis. After the infection of adenovirus expressing p53 or LacZ as a control, shRNA-treated populations were analyzed by microarray. We identified shRNAs that were significantly decreased in p53-infected cells compared with control cells. Among these shRNAs, shRNA-58335 was markedly decreased in both cancer cell lines tested. shRNA-58335 enhanced p53-related apoptosis *in vitro* and augmented the inhibitory effect of adenoviral p53 transduction on tumor growth *in vivo.* Furthermore, the enhanced apoptotic response by shRNA-58335 was also confirmed by treatment with PRIMA-1, which reactivates mutant p53, instead of adenoviral p53 transduction. We found that shRNA-58335 evokes the apoptotic response following p53 transduction or functional restoration of p53 with a small molecule drug in cancer cells resistant to p53-induced apoptosis. The combination of p53 restoration and RNAi-based drugs is expected to be a promising novel cancer therapy.

## INTRODUCTION

p53 is one of the most important tumor suppressor genes. In approximately half of all human cancers, p53 is inactivated as a direct result of mutations in the p53 gene [1]. Furthermore, mutation or deletion of p53 is related to poor prognosis and resistance to chemotherapy and radiation [2]. Therefore, vector-mediated gene transfer of p53 has been viewed as a potentially effective cancer therapy. However, gene transfer of p53 does not always result in good therapeutic outcomes in all cancers [3, 4]. The inactivation of apoptotic machinery is considered as one of the reasons that p53 transduction fails to induce apoptosis in cancers. In such cancers, p53 status does not necessarily correlate with the responsiveness to chemotherapy [5].

The activation of p53 is induced by a variety of cell stresses, such as DNA damage, oncogene activation, spindle damage and hypoxia. Activated p53 transactivates not only coding genes but also non-coding RNAs, including microRNAs (miRNAs) and large intergenic non-coding RNAs (lincRNAs), many of which are involved in DNA repair, cell cycle arrest and apoptosis [6, 7]. Although the precise mechanisms that regulate whether a cell undergoes cell cycle arrest or apoptosis are unclear, cell cycle arrest induced by p53 is mediated primarily through the induction of the expression of p21 [8], which is involved in restoring genomic integrity by functioning in an anti-apoptotic manner [9]. Thus, the suppression of p53-induced expression of p21 may lead to the preferential induction of apoptosis rather than cell cycle arrest following p53 activation. In our previous study, we constructed a replication-deficient recombinant adenovirus that encoded co-cistronic p53 and artificial microRNAs targeting endogenous p21, resulting in simultaneous p53 expression and the suppression of p21 induction. In colorectal and hepatocellular carcinoma cells, infection with the adenovirus vector augmented apoptosis and suppressed tumor growth compared with an adenovirus that expressed p53 alone *in vitro* and *in vivo* [10]. Thus, the combination of p53 and RNAi was shown to be a potential novel cancer therapy.

To discover factors that modulate p53 pathways, we attempted to identify RNAi sequences that enhance p53-induced apoptosis. In this study, we screened a genome-wide lentiviral shRNA library in cancer cells resistant to p53-related apoptosis. We identified specific shRNAs that were significantly decreased in p53-infected cells but not in control cells. These results indicated that specific shRNAs evoked the apoptotic response following p53 transduction or functional restoration of p53 in cancer cells resistant to p53-induced apoptosis, suggesting that the combination of p53 restoration and treatment with specific shRNAs may be an effective cancer therapy.

## RESULTS

### Genome-wide shRNA screening in p53-resistant cancer cells

**Figure 1:**
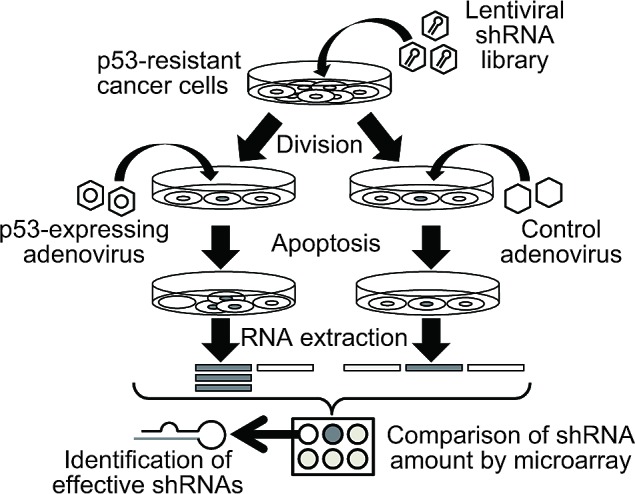
Schematic representation of the array-based shRNA library screening process p53-resistant cancer cells were infected with a pooled lentiviral shRNA library. After lentiviral infection, the cells were infected with adenovirus expressing p53 or LacZ. The shRNA population was analyzed by a cDNA microarray-based approach.

To identify shRNAs that can relieve resistance to p53-induced apoptosis in cancer cells, we screened a genome-wide lentiviral shRNA library in liver cancer Huh-7 cells and pancreatic cancer Panc-1 cells, as summarized in Figure 1A and briefly described below. The Huh-7 and Panc-1 cell lines have mutated p53 and a weak apoptotic response following wild-type p53 transduction. The human shRNA library, comprising approximately 200,000 shRNA sequences targeting 47,400 human mRNA transcripts, was packaged into lentivirus particles and pooled. First, we infected the cell lines with the pooled lentiviral shRNA library. Seventy-two hours after lentiviral infection, the cells were infected with adenovirus expressing p53. Forty-eight hours after adenoviral infection, total mRNA was extracted, and mRNA, including shRNA sequences derived from lentiviral vectors, was amplified by PCR. In this shRNA library, each lentiviral vector shRNA sequence corresponds to a probe sequence found on an Affymetrix microarray (GeneChip). Therefore, the shRNA populations can be quantified by cDNA microarray to determine the expression levels of each shRNA; as a reference, the shRNA population in each cell line infected with adenovirus expressing LacZ was also analyzed.

### Identification of shRNA-decreased populations in p53-transduced cells

**Figure 2:**
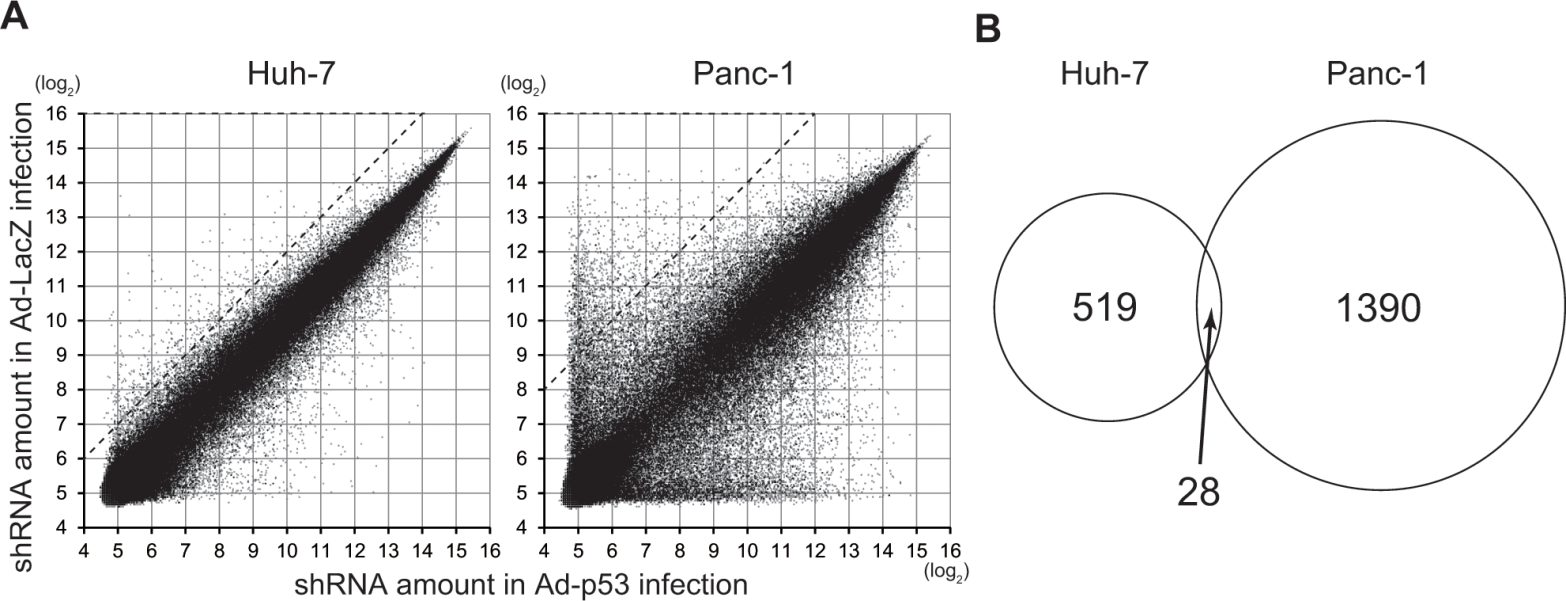
Analysis of the shRNA library screen in p53-transduced cells (A) shRNA populations were quantified by cDNA microarray in p53-transduced Huh-7 and Panc-1 cells. The amount of each shRNA in p53-transduced cells was plotted against the amount of the shRNA in LacZ-transduced control cells as log_2_. In Huh-7 cells, 547 shRNAs decreased more than 4-fold in p53-transduced cells compared with control cells. In Panc-1 cells, 1418 shRNAs decreased more than 16-fold in p53-transduced cells compared with control cells (areas encircled by dashed lines). (B) The overlap of the selected shRNAs in Huh-7 and Panc-1 cells is displayed in a Venn diagram.

We identified the shRNAs that were significantly decreased in p53-infected cells compared with control cells (Fig. 2A). Several shRNAs evoked the apoptotic response following p53 transduction in cancer cells resistant to p53-induced apoptosis. In Huh-7 cells, 547 shRNAs were decreased more than 4-fold in cells with p53 transduction compared with control cells. In Panc-1 cells, 1418 shRNA were decreased more than 16-fold in cells with p53 transduction compared with control cells (Fig. 2A, areas encircled by dashed line). Furthermore, 28 shRNAs were commonly decreased in both cell lines (Fig. 2B, Supplementary Table S1). Among these shRNAs, shRNA-58335 in the presence of p53 was markedly decreased (66-fold in Huh-7 cells and 436-fold in Panc-1 cells). Therefore, we further analyzed shRNA-58335.

### Enhancement of p53-induced apoptosis by shRNA-58335

**Figure 3:**
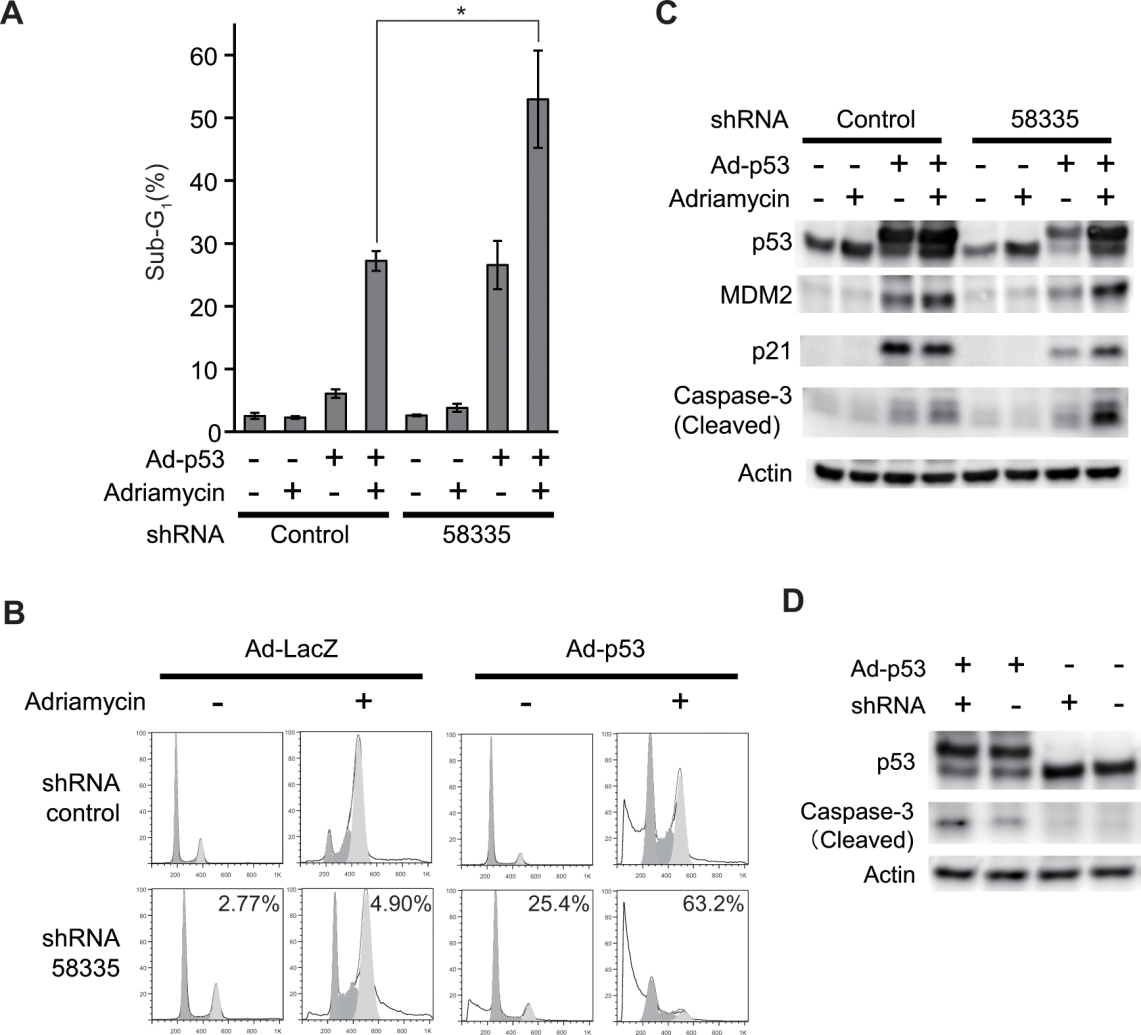
Effect of shRNA-58335 on p53-induced apoptosis (A) Huh-7 cells were stably infected with lentivirus expressing shRNA-58335 or a control sequence. These cells were then infected with an adenovirus expressing p53 (Ad-p53: +) or LacZ (Ad-p53: -) as a control at an MOI of 200. Twenty-four hours after infection, the cells were treated with adriamycin (0.5 μg/ml) or not. Forty-eight hours after treatment, the cells were analyzed by flow cytometry. The percentage of cells in the sub-G_1_ phase is indicated. Error bars indicate the S.E. * indicates a p value < 0.05 by a *t*-test. (B) Representative flow cytometry data in Huh-7 cells infected with Ad-p53. The percentage of cells in the sub-G_1_ phase is indicated. (C) Under the same conditions used in (A), total cell lysates were extracted and analyzed by Western blot with the indicated antibodies. (D) SW480 cells were infected with lentivirus expressing shRNA-58335 (shRNA: +) or a control sequence (shRNA: -). These cells were then infected with an adenovirus expressing p53 (Ad-p53: +) or LacZ (Ad-p53: -) as a control at an MOI of 200. Forty-eight hours after infection, total cell lysates were extracted and analyzed by Western blot with the indicated antibodies.

Next, we stably infected lentivirus expressing shRNA-58335 or a control sequence into Huh-7 cells and quantified p53-induced apoptosis by evaluating the sub-G_1_ population. In these cells, p53 transduction induced a strong apoptotic response in shRNA-58335-infected cells compared with control cells (Fig. 3A, B). Additionally, treatment of adriamycin significantly enhanced the apoptotic response (Fig. 3A, B). Increased caspase-3 cleavage, which serves as another indicator of apoptosis, was also observed by western blotting (Fig. 3C). In colorectal cancer SW480 cells, which also have mutated p53 and a weak apoptotic response following p53 transduction, shRNA-58335 sensitized the p53-induced apoptotic response (Fig. 3D). These results indicated that shRNA-58335 improved the apoptotic response after p53 transduction in other cancer cell lines.

### Inhibition of tumor growth by p53 transduction with shRNA-58335 *in vivo*

**Figure 4:**
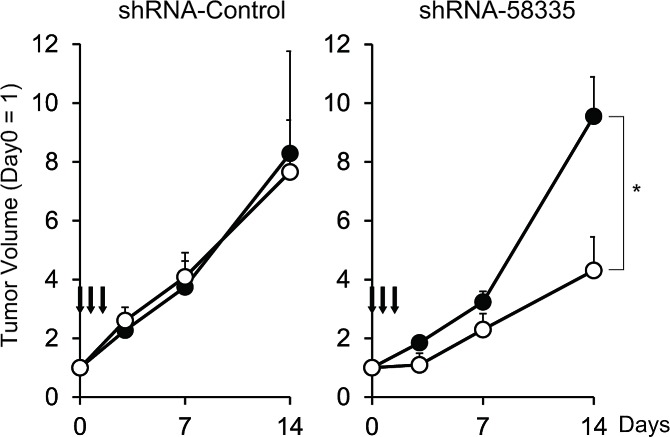
Therapeutic effect of shRNA-58335 *in vivo* Huh-7 cells stably infected with lentivirus expressing shRNA-58335 or control sequences were injected s.c. into nude mice. When the tumor volume reached 100 mm^3^, adenovirus expressing p53 (Ad-p53) or LacZ as a control (Ad-LacZ) was injected directly into the tumors at days 0, 1 and 2 (indicated by arrows). Ad-p53, open circle; Ad-LacZ, closed circle. The data represent the average volume of three independent tumors injected with adenovirus. The volume of each tumor is expressed relative to the volume at day 0, which was set as 1. Error bars indicates S.E. * indicates a p value < 0.05 by a *t*-test.

To confirm whether the improved p53-induced apoptotic response *in vitro* was correlated with a therapeutic effect *in vivo*, we examined the tumor suppressive effect of p53 transduction in a xenograft model of tumorigenesis. shRNA-infected Huh-7 cells were injected s.c. into nude mice. When tumor volume reached a consistent size, adenovirus expressing p53 or LacZ was injected directly into the tumor at days 0, 1 and 2 (Fig. 4, arrow). In control shRNA-infected Huh-7 cells, the injection of Ad-p53 did not affect tumor volume (Fig. 4, left). Conversely, in shRNA-58335-infected Huh-7 cells, tumor volume was significantly decreased by injection of Ad-p53 compared with Ad-LacZ (Fig. 4, right). These results suggest that combined treatment with p53 and a specific shRNA may be an effective cancer therapy *in vivo*.

### Enhancement of PRIMA-1-induced apoptosis by shRNA-58335

**Figure 5:**
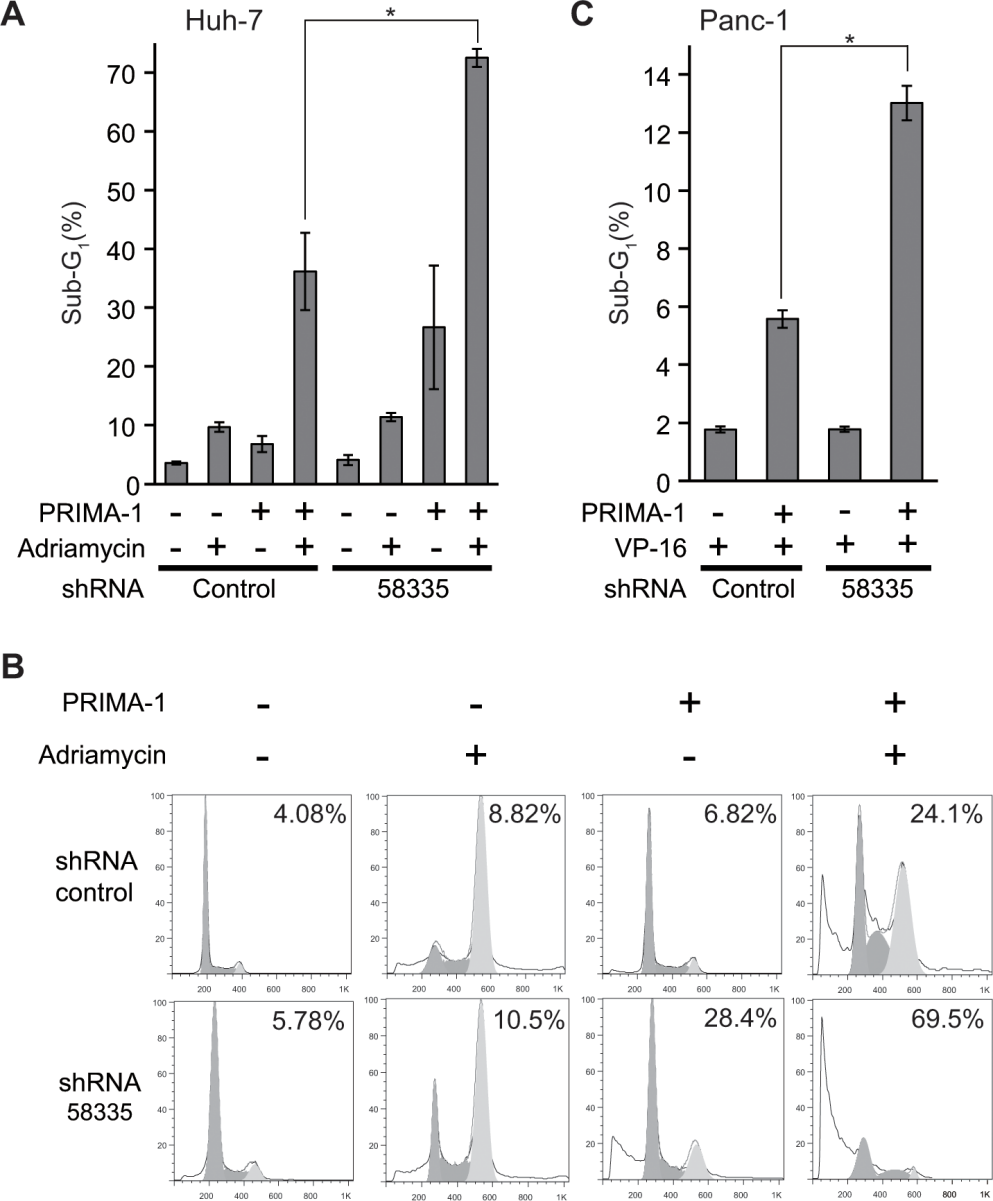
Effect of shRNA-58335 on apoptosis with the functional restoration of p53 with PRIMA-1 (A) Huh-7 cells stably infected with lentivirus expressing shRNA-58335 or control sequences were treated with PRIMA-1 (200 μM) and/or adriamycin (0.5 μg/ml). Seventy-two hours after treatment, the cells were analyzed by flow cytometry. The percentage of cells in the sub-G_1_ phase is indicated. (B) Representative flow cytometry data in Huh-7 cells treated with PRIMA-1 and adriamycin. The percentage of cells in the sub-G_1_ phase is indicated. (C) Panc-1 cells were stably infected with lentivirus expressing shRNA-58335 or a control sequence. These cells were treated with VP-16 (300 μM) in the presence or absence of PRIMA-1 (200 μM). Forty-eight hours after treatment, the cells were analyzed by flow cytometry. The percentage of cells in the sub-G_1_ phase is indicated. In (A) and (C), error bars indicate the S.E. * indicates a p value < 0.05 by *t*-test.

p53 transduction by adenoviral vector is difficult to deliver to cancer tissues *in vivo*. Previous report indicated that histone deacetylase inhibitors reactivated mutant p53 [12]. Recently, PRIMA-1 was identified as a low-molecular-weight compound that restores the sequence-specific DNA binding and active conformation of mutant p53 proteins [13] and demonstrated positive data in phase 1/2 clinical trials of hematologic malignancies and prostate cancer [14]. Therefore, we replaced adenoviral p53 transduction with PRIMA-1 treatment to activate p53, performed as in Fig. 3A. As observed in Fig. 3A, the combinatorial treatment of PRIMA-1 and adriamycin induced a strong apoptotic response in shRNA-58335-infected cells compared with control-shRNA-infected cells (Fig. 5A, B). Furthermore, we evaluated the apoptotic effect on Panc-1 cells by shRNA-58335 and PRIMA-1. Adriamycin treatment alone did not induce an apoptotic response in Panc-1 cells (data not shown). Therefore, we treated Panc-1 cells with VP-16 (etoposide) and quantified apoptosis by evaluating the sub-G_1_ population (Fig. 5C). The combinatorial treatment of PRIMA-1 and VP-16 significantly enhanced the apoptotic response in shRNA-58335-infected cells compared with control-shRNA-infected cells. These results demonstrated that shRNA-58335 effectively enhanced the p53-induced apoptotic response not only by p53 transduction but also by the functional restoration of p53 with small molecule treatment.

### Altered gene expression pattern by shRNA-58335 in p53-transduced cells

According to the NCBI BLAST search (http://blast.ncbi.nlm.nih.gov/), the seed sequence of shRNA-58335 is perfectly matched only to AP2γ (TFAP2C) among all human transcripts in the RefSeq database. AP2γ belongs to the AP-2 family of transcription factors and overexpressed in human breast cancers and the overexpression related with reduced patient survival [15]. A recent report demonstrated that AP2γ is a key transcriptional regulator for maintaining the luminal phenotype in human breast carcinoma [16]. AP2γ is also a direct transcriptional target of p53 [17]. We confirmed a statistically significant but slight decrease in AP2γ mRNA levels by shRNA-58335 (Supplementary Figure S1). However, we were unable to detect AP2γ protein using Western blotting. Therefore, it remains controversial whether shRNA-58335 works through the suppression of AP2γ.

To explore the effect of shRNA-58335 on p53-induced apoptosis, we evaluated gene expression by cDNA microarray analysis in p53-transduced Huh-7 cells. We found that the expression of 350 genes was decreased more than 2-fold in shRNA-58335-infected Huh-7 cells compared with control-shRNA infected cells (Supplementary Table S2). Interestingly, gene ontology analysis revealed that these genes were significantly associated with cell cycle progression and cell division (Table 1). These results indicate that shRNA-58335 evoked an apoptotic response following p53 transduction by modulating the gene expression associated with the process of cell division.

**Table 1:** Gene ontology analysis of 350 genes decreased by shRNA-58335 in p53-transduced Huh-7 cells

Term	P value
mitosis	9.91×10^−32^
nuclear division	9.91×10^−32^
M phase of mitotic cell cycle	1.97×10^−31^
organelle fission	4.57×10^−31^
M phase	8.39×10^−30^
cell cycle	1.86×10^−29^
mitotic cell cycle	7.17×10^−29^
cell cycle phase	5.76×10^−28^
cell cycle process	2.35×10^−26^
cell division	1.83×10^−22^
chromosome segregation	1.07×10^−12^
microtubule-based process	5.55×10^−12^
spindle organization	1.46×10^−10^
microtubule cytoskeleton organization	3.46×10^−8^
mitotic cell cycle checkpoint	4.80×10^−8^
regulation of cell cycle	1.54×10^−7^
cell cycle checkpoint	1.80×10^−7^
spindle checkpoint	2.05×10^−7^
regulation of mitotic cell cycle	4.11×10^−7^
mitotic spindle organization	7.56×10^−7^
protein-DNA complex assembly	1.86×10^−6^
mitotic sister chromatid segregation	4.80×10^−6^
sister chromatid segregation	5.67×10^−6^
regulation of mitotic metaphase/anaphase transition	6.18×10^−6^
mitotic cell cycle spindle assembly checkpoint	7.08×10^−6^
negative regulation of mitotic metaphase/anaphase transition	7.08×10^−6^
cytoskeleton organization	8.02×10^−6^
chromosome organization	9.36×10^−6^
cellular macromolecular complex subunit organization	9.53×10^−6^
negative regulation of mitosis	1.05×10^−5^
negative regulation of nuclear division	1.05×10^−5^
microtubule-based movement	1.12×10^−5^
DNA packaging	1.49×10^−5^
organelle localization	1.84×10^−5^
chromosome localization	2.82×10^−5^
establishment of chromosome localization	2.82×10^−5^
cellular macromolecular complex assembly	4.28×10^−5^
nucleosome assembly	8.16×10^−5^
regulation of cell cycle process	8.63×10^−5^

## DISCUSSION

We demonstrated shRNAs that enhanced p53-related apoptosis by screening a genome-wide shRNA library. Conventional shRNA library screens have shown shRNAs that resist p53-induced apoptosis through the presence of p53-resistant cellular colonies [18]. However, this method is not able to identify shRNAs that enhance p53-related apoptosis because cells that include such shRNAs are lost. Recently, array-based shRNA screening was developed, allowing the direct quantification of the amount of each shRNA. We applied this new method here and successfully identified shRNAs that enhanced p53-related apoptosis.

Although the shRNA library used in this study included approximately 200,000 shRNA sequences targeting 47,400 human mRNA transcripts, the knockdown efficacy of each shRNA has not been validated. Therefore, the screened shRNAs, the sequences of which match specific transcripts, are not necessarily able to effectively knockdown their target genes. In fact, only weak suppression of the *TFAP2C* gene by shRNA-58335 was confirmed, although the seed sequence of shRNA-58335 completely matched the AP2γ mRNA sequence (Supplementary Figure 1). Furthermore, populations of other shRNAs that target AP2γ mRNA (shRNA-58334, 58336 and 58337) were not significantly decreased in p53-infected Huh-7 or Panc-1 cells compared with LacZ-infected control cells. However, shRNA-58335 effectively enhanced the p53-induced apoptotic response not only by p53 transduction (Fig. 3, 4) but also by the functional restoration of p53 with small molecule PRIMA-1 (Fig. 5). These results may suggest that shRNA-58335 operates as a molecular modulator in p53-related apoptosis through other unknown mechanisms rather than through the knockdown of the *TFAP2C* gene. Our microarray analysis revealed that shRNA-58335 with p53 transduction modulates gene expression associated with the process of cell division (Table 1), whereas no genes were significantly decreased more than twofold in shRNA-58335-infected cells without p53 transduction compared with control-shRNA-infected cells. These results also support our conclusion that shRNA-58335 does not knockdown any specific genes. However, the molecular mechanism of shRNA-58335-mediated enhancement of p53-related apoptosis is unclear. Further functional analysis should be performed in future studies.

We demonstrated that the apoptotic response induced by adenoviral p53 transduction was enhanced by shRNA-58335 in cancer cells resistant to p53-induced apoptosis (Fig. 3, 4). However, the clinical application of adenoviral vectors is presently limited because adenoviral vectors must be administered locally [4]. Therefore, we replaced adenoviral p53 transduction with PRIMA-1 treatment to reactivate mutant p53 [13] and successfully confirmed the enhancement of an apoptotic response by PRIMA-1 with shRNA-58335 treatment (Fig. 5). PRIMA-1 can be intravenously administered in human clinical studies [14]. Furthermore, non-viral delivery systems, such as nanoparticles, for RNAi-based drug treatments are also being developed and are in various stages of clinical investigation [19, 20].

Most of clinically used chemotherapeutic agents cause DNA damage and target dividing cells in S or M phase. Therefore, chemotherapeutic agents not only kill cancer cells but also injury normal dividing cells, resulting in adverse effects of cancer treatment. In order to protect normal cells from chemotherapeutic agents, pretreatment of drugs which induce cell cycle arrest in normal cells, so-called “cyclotherapy” is considered as an effective therapy [21, 22]. Recent reports suggest that the pretreatment of small-molecule p53 activators such as nutlin-3 protect normal cells from chemotherapeutic agents [23, 24]. The combination of p53 restoration, RNAi-based drugs and p53-based cyclotherapy is expected to be a promising novel cancer therapy in the near future.

## MATERIALS AND METHODS

### Cell culture

The hepatic cancer Huh-7 cell line, the pancreatic cancer Panc-1 cell line and the colorectal cancer SW480 cell line were purchased from the American Type Culture Collection. Huh-7, Panc-1 and SW480 cells were cultured in DMEM, RPMI-1640 and Leibovitz L-15 medium with 10% FCS, respectively. The construction, purification and infection of replication-deficient recombinant adenoviruses containing p53 (Ad-p53) or the bacterial lacZ gene (Ad-LacZ) have been described previously [11].

### shRNA library screening

shRNA library screening was performed according to the manufacturer's protocol. Briefly, 3×10^6^ Huh-7 and Panc-1 cells were plated on a 10-cm plate. Twenty-four hours after plating, the cells were infected with lentivirus particles from the GeneNet siRNA Library (System Biosciences). Seventy-two hours after lentiviral infection, Ad-p53 or Ad-LacZ was infected into these cells at an MOI of 50. Forty-eight hours after adenoviral infection, total RNA was extracted with TRIZol (Invitrogen). cDNA was synthesized from extracted RNAs. siRNA inserts were amplified from cDNA using two rounds of PCR with biotinylated primers. PCR products were treated with lambda exonuclease to remove the sense non-biotinylated strand. Ten micrograms of biotinylated samples was hybridized to an Affymetrix GenChip microarray (HG-U133+ 2.0). The obtained data were analyzed with GeneNet siRNA Library Data Analysis Software (System Biosciences).

### Establishment of cell lines stably expressing shRNAs

Huh-7 and Panc-1 cells were infected with lentivirus (pSIH-H1-puro) expressing shRNA-58335 (5'-TGGCGGCCCAGCAACTGTGTAAAGA ATCTTCCTGTCAGAATTCTTTACACAGTTGCTGG GCCGCCA-3') or luciferase control shRNA (5'-GTGCGTTGTTAGTACTAATCCTATTTGT GAAGCAGATGAAATAGGGTTGGTACTAGCAAC GCAC-3'). Twenty-four hours after infection, these cells were cultured with media containing puromycin (2 μg/ml in Huh-7 cells and 4 μg/ml in Panc-1 cells, respectively) and resistant cells were selected.

### Antibodies and reagents

Adriamycin and VP-16 were purchased from Sigma. PRIMA-1 was purchased from Cayman Chemical. The anti-p53 (DO-1), anti-MDM2 (SMP14) and anti-p21 (F-5) mouse antibodies were purchased from Santa Cruz Biotechnology. The anti-actin mouse antibody was purchased from Millipore, and the anti-caspase-3 (8G10) rabbit antibody was purchased from Cell Signaling Technology.

### Western blot analysis

Total cell lysate was extracted at 4°C with RIPA buffer (150 mM NaCl, 1% NP40, 0.5% sodium deoxycholate, 0.1% SDS and 50 mM Tris HCl, pH 8.0). The samples were fractionated by SDS-PAGE and transferred onto Immobilon-P membranes (Millipore). Immunoreactive proteins were detected using enhanced chemiluminescence (ECL) (GE Healthcare).

### Flow cytometry

Cells (1×10^6^) were plated on 6-well plates. After infection or treatment, the cells were harvested by trypsinization and pelleted by centrifugation. Pelleted cells were fixed in 90% cold ethanol, treated with RNase A (500 units/ml) and then stained with propidium iodide (50 mg/ml). The samples were analyzed on a FACSCalibur flow cytometer (BD Bioscience). The experiments were repeated at least three times, and 50,000 events were analyzed for each sample. Data were analyzed using FlowJo software (Tree Star).

### Animal models

All animals were maintained under specific pathogen free conditions and treated in accordance with guidelines set by the Animal Care and Use Committee of Sapporo Medical University. To evaluate the effects of treating established tumors, 24 female BALB/c nude mice were injected subcutaneously (s.c.) with 2×10^6^ Huh-7 cells infected with lentiviral shRNA-58335 or control vector into both flanks. When the tumor size reached 100 mm^3^, the mice received a direct intratumoral injection of 1×10^9^ p.f.u. (in 100 μl of PBS) of the indicated adenovirus a total of three times on days 0, 1 and 2. Three mice were used for each treatment group. Tumor formation in the mice was monitored for up to 2 weeks. The tumor volume was calculated using the equation V (mm^3^) = a×b^2^/2, where “a” represents the largest dimension and “b” is the perpendicular diameter.

### Microarray analysis

Huh-7 cells stably expressing shRNA-58335 or control shRNA were infected with adenovirus expressing p53 (Ad-p53) or LacZ (Ad-LacZ) as control at 200 moi. Seventy-two hours after infection, total RNA was extracted by RNeasy mini kit (QIAGEN) according to manufacturer's protocol. Total RNA was labeled with Cy3, hybridized to a microarray (Agilent SurePrint G3 Human GE) and scanned by Agilent SureScan according to the manufacturer's protocols. The obtained data were normalized using Limma (R package). The microarray data were deposited in the NCBI Gene Expression Omnibus (GEO, accession number: GSE56156).

### SUPPLEMENTAL MATERIAL AND FIGURE



### SUPPLEMENTAL MATERIAL AND TABLES


