# Supplementary material for: Array-based genome-wide RNAi screening to identify shRNAs that enhance p53-related apoptosis in human cancer cells
Source: Oncotarget. 2014 Jul 27;5(17):7540–8. doi: 10.18632/oncotarget.2272 (PMC4202142; doi:10.18632/oncotarget.2272)
Supplement: Supplementary file 1 [file oncotarget-05-7540-s001.pdf]

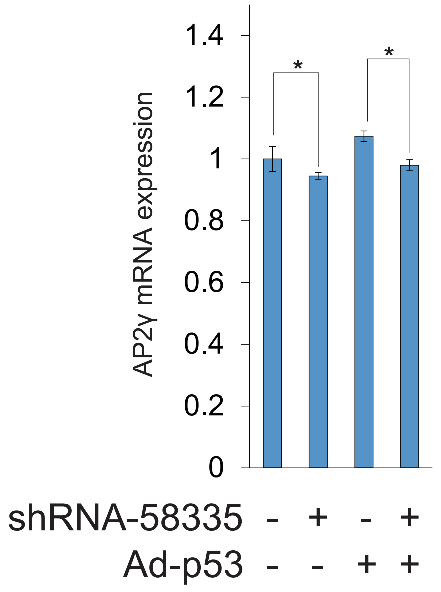

Supplemental Figure S1. Effect of shRNA-58335 on AP2γ expression.

Panc-1 cells stably infected with lentivirus expressing shRNA-control (shRNA-58335 -) or shRNA-58335 were infected with adenovirus expressing LacZ (Ad-p53 -) or p53 (Ad-p53 +). 24 h after infection, AP2γ mRNA expression was quantified via RT-qPCR with TaqMan Gene Expression Assays (Hs00231476_m1, Life Technologies).

The averages of three experiments are indicated; shRNA-control + Ad-LacZ = 1.

Error bars indicate S.D. * indicates p values < 0.05 according to *t*-tests.
